# Red Blood Cell Lysis Pretreatment Can Significantly Improve the Yield of Treponema pallidum DNA from Blood

**DOI:** 10.1128/spectrum.05198-22

**Published:** 2023-05-24

**Authors:** Zi-Han Wei, Lin Xie, Yong-Jing Wang, Jiang-Xing Zhuang, Jian-Jun Niu, Li-Li Liu

**Affiliations:** a Center of Clinical Laboratory, Zhongshan Hospital of Xiamen University, School of Medicine, Xiamen University, Xiamen, China; b Institute of Infectious Disease, School of Medicine, Xiamen University, Xiamen, China; c Xiamen Clinical Laboratory Quality Control Center, Xiamen, China; d Fujian Provincial Key Laboratory of Neurodegenerative Disease and Aging Research, Institute of Neuroscience, School of Medicine, Xiamen University, Xiamen, China; Quest Diagnostics

**Keywords:** PCR, sample preparation, blood, blood component, DNA yield, *Treponema pallidum*, red blood cell lysis

## Abstract

PCR can be a supplement to Treponema serological testing. However, its sensitivity is not satisfactory for blood sample testing. The aim of this study was to investigate whether pretreatment with red blood cell (RBC) lysis could enhance the yield of Treponema pallidum subsp. *pallidum* DNA extraction from blood. We developed and verified the efficacy of a quantitative PCR (qPCR) assay that utilizes TaqMan technology to specifically detect T. pallidum DNA by targeting the *polA* gene. Simulation media with 10^6^ to 10^0^ treponemes/mL were prepared in normal saline (NS), whole blood, plasma, and serum, and RBC lysis pretreatment was performed on a portion of whole blood. Then, blood samples drawn from 50 syphilitic rabbits were divided in parallel into five groups, labeled whole blood, whole blood/lysed RBCs, plasma, serum, and blood cells/lysed RBCs. DNA extraction and qPCR detection were performed. The detection rate and copy number were compared among different groups. The *polA* assay showed good linearity and an excellent amplification efficiency of 102%. In the simulated blood samples, the detection limit of the *polA* assay reached 1 × 10^2^ treponemes/mL in whole blood/lysed RBCs, plasma, and serum. However, the detection limit was only 1 × 10^4^ treponemes/mL in NS and whole blood. Among the blood samples from syphilitic rabbits, whole blood/lysed RBCs showed the best detection rate (82.0%), while the detection rate for whole blood was only 6%. The copy number of whole blood/lysed RBCs was higher than that of whole blood. RBC lysis pretreatment can significantly improve the yield of T. pallidum DNA from whole blood, and the yield is better than that from whole blood, plasma, serum, and blood cells/lysed RBCs.

**IMPORTANCE** Syphilis is a sexually transmitted disease caused by T. pallidum that can spread into the blood. T. pallidum DNA can be detected in blood by PCR but with low sensitivity. Few studies have applied RBC lysis pretreatment to blood T. pallidum DNA extraction. This study shows that the detection limit, detection rate, and copy number of whole blood/lysed RBCs were better than those of whole blood, plasma, and serum. After RBC lysis pretreatment, the yield of low concentrations of T. pallidum DNA was improved, and the low sensitivity of blood-based T. pallidum PCR was improved. Therefore, whole blood/lysed RBCs are the ideal sample for acquiring blood T. pallidum DNA.

## INTRODUCTION

Syphilis is a sexually transmitted disease caused by Treponema pallidum subsp. *pallidum*. Syphilis has been persistent for hundreds of years, and its diagnosis remains a challenge (1). Serological testing is the mainstay of syphilis diagnosis. The interpretation of serological test results can pose a challenge for certain types of syphilis, including early primary syphilis, congenital syphilis, and syphilis concomitant with HIV infection. This is because of the absence or delay of serological responses, which may hinder accurate diagnosis and management of these specific syphilis subtypes (2–4). PCR is considered highly sensitive for T. pallidum DNA detection in swab samples but less sensitive in blood samples (5). Swab samples, taken from chancres and skin rashes, which can heal spontaneously, are not always obtained. Accordingly, blood samples have become an alternative. Since it is still not clear when and to what extent T. pallidum spreads into the blood, the detection rate of T. pallidum DNA in blood is not satisfactory (6–8). To improve the sensitivity of blood-based PCR tests, many researchers have focused on samples from different sources and collected at different stages, different DNA extraction protocols, and different PCR targets and methods (9). PCR sensitivities are different for whole blood (WB), plasma, peripheral blood mononuclear cells (PBMCs), serum, and blood clots. However, there is still controversy regarding the best blood component for the detection of T. pallidum DNA (5, 10). Salazar et al. (7) and Tipple et al. (8) found that whole blood was most suitable, while Castro et al. (11) and Wang et al. (12) thought that plasma was the best, and Kouznetsov et al. (13) and Grange et al. (5) considered PBMCs to be a more reliable source. Zhu et al. (14) found that the T. pallidum DNA extraction efficiency from blood clots was very poor. Whole-blood pretreatment with red blood cell (RBC) lysis buffer has been used for PCR detection of blood pathogens (15, 16). Our previous study (14) used RBC lysis pretreatment for blood T. pallidum DNA extraction, but that study focused on high-concentration simulation samples and did not compare whole blood/lysed RBCs (WBL) with other blood components. Since T. pallidum tends to adhere to the extracellular matrix (17), we considered blood cells to be a better choice than other blood components for detecting T. pallidum DNA. The main difficulty in detecting T. pallidum in blood is that the concentration of T. pallidum is very low (12). Thus, loss of T. pallidum DNA could easily occur in the DNA extraction process due to DNA binding failure. On the other hand, unremoved hemoglobin will inhibit PCR, leading to false-negative PCR results (18). We hypothesized that RBC lysis pretreatment before DNA extraction could overcome the disadvantages of whole-blood extraction, thus improving the detection rate of syphilitic blood.

In this study, we developed a TaqMan-based real-time quantitative PCR (qPCR) assay targeting the *polA* gene, which was subsequently employed in a range of *in vitro* simulation experiments, as well as in infected rabbit blood, to detect T. pallidum DNA. The purpose of this study was to assess whether red blood cell (RBC) lysis pretreatment could improve the yield of T. pallidum DNA from blood by improving the process.

## RESULTS

### Performance of the *polA* qPCR assay.

**FIG 1:**
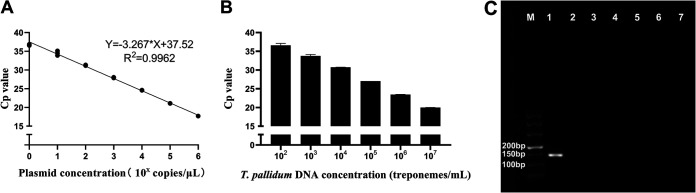
Standard curve, limit of detection, and electrophoresis results of the *polA* assay. (A) Standard curve of the *polA* assay. Plasmids were serially diluted 10-fold to obtain a range of concentrations from 1 × 10^6^ copies/μL to 1 × 10^0^ copies/μL. (B) Limit of detection of the *polA* assay, with data represented as the mean ± SD of three technical replications. T. pallidum DNA was serially 10-fold diluted to obtain a range of concentrations from 1 × 10^7^ treponemes/mL to 1 × 10^0^ treponemes/mL; 1 × 10^2^ treponemes/mL is equal to 1.93 × 10^3^ copies/mL, 1 × 10^3^ treponemes/mL is equal to 1.42 × 10^4^ copies/mL, 1 × 10^4^ treponemes/mL is equal to 1.19 × 10^5^ copies/mL, 1 × 10^5^ treponemes/mL is equal to 1.60 × 10^6^ copies/mL, 1 × 10^6^ treponemes/mL is equal to 2.04 × 10^7^ copies/mL, and 1 × 10^7^ treponemes/mL is equal to 2.36 × 10^8^ copies/mL. (C) Specificity of the *polA* assay detected by electrophoresis. The base length of the *polA* target band is 143 bp. Lane M, 500 bp marker; lane 1, T. pallidum DNA; lane 2, Chlamydia trachomatis DNA; lane 3, *Herpes simplex virus* DNA; lane 4, *Human papillomavirus* DNA; lane 5, Neisseria gonorrhoeae DNA; lane 6, human DNA; lane 7, rabbit DNA. Abbreviations: Cp, cross point; *R*^2^, coefficient of determination.

The *polA* assay showed good linearity, and the slope was −3.267, indicating an amplification efficiency of 102%. The assay detected concentrations as low as 10^0^ copies/μL plasmid. Standard curves are shown in Fig. 1A. When applied to T. pallidum DNA testing, the fluorescent signal was clearly visible when the dilution reached 1/10^5^. Thus, 1 × 10^2^ treponemes/mL was determined to be the lower limit of reliable detection (Fig. 1B).

Demonstrating the specificity of the established *polA* qPCR method, the electrophoresis results of the amplified products were consistent with the PCR results. This assay was able to detect T. pallidum DNA and did not detect DNA from other sexually transmitted organisms, such as Chlamydia trachomatis, *Herpes simplex virus* (HSV), *Human papillomavirus* (HPV), and Neisseria gonorrhoeae, or from healthy humans and normal rabbits, as shown in Fig. 1C. DNA sequencing results revealed that the amplicon had 100% sequence homology with its target fragment (data not shown).

### Analysis of blood fractions spiked with T. pallidum *ex vivo*.

**FIG 2:**
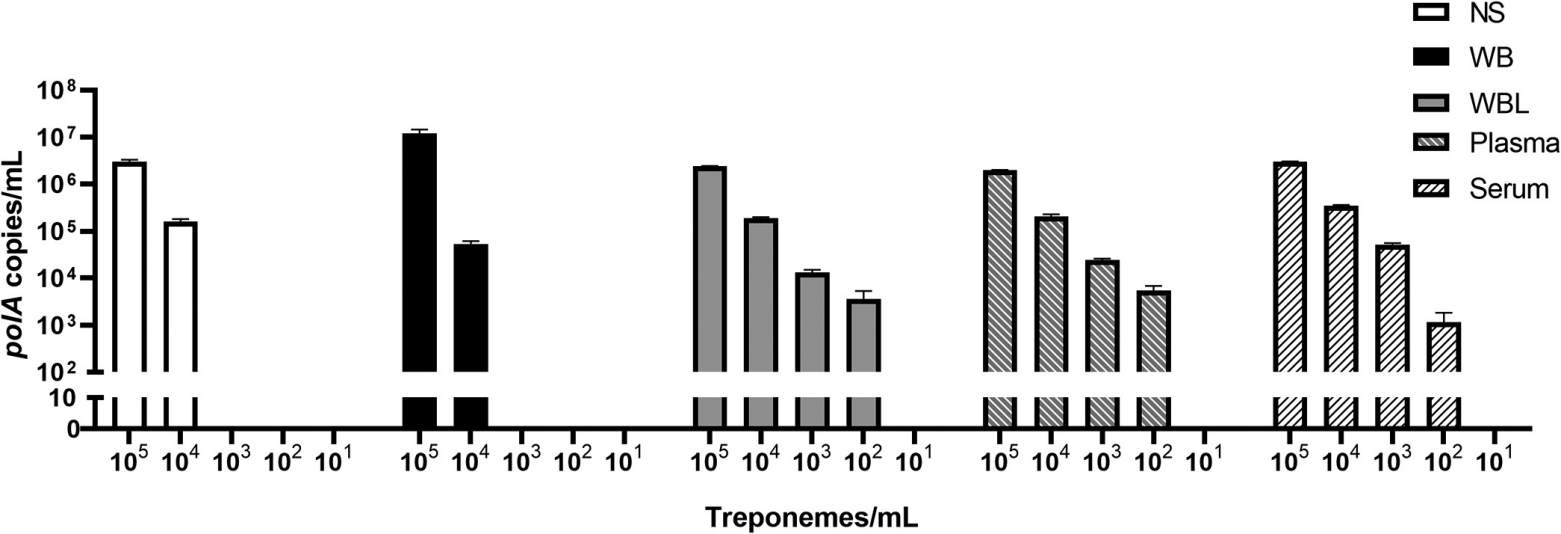
Assay results for gradient concentrations of T. pallidum in different simulated media. Data are represented as the mean ± SD of three technical replications. The copy number was calculated from the standard curve. The minimum detection limit was 1 × 10^2^ treponemes/mL in the WBL, plasma, and serum groups. The minimum detection limit was 1 × 10^4^ treponemes/mL in the NS and WB groups. The lower concentration and negative medium controls were all negative. Abbreviations: NS, normal saline; WB, whole blood; WBL, whole blood/lysed RBCs.

Five kinds of simulation media (normal saline [NS], WB, WBL, plasma, and serum) were prepared with ~1.0 × 10^5^ to 1.0 × 10^0^ treponemes/mL. As shown in Fig. 2, when the concentration of T. pallidum gradually decreased, the copy number detected in different media gradually decreased. Although template loss may have occurred during DNA extraction from the WBL, plasma, and serum groups, the T. pallidum DNA detection limit for these groups still reached 1 × 10^2^ treponemes/mL. In the NS and WB groups, however, 1 × 10^4^ treponemes/mL was the minimum detection limit. In the two kinds of whole-blood dilution experiments (simulated whole blood with NS [SBN] and whole blood diluted with NS [BDN]), 1 × 10^2^ treponemes/mL was the lowest concentration that was detectable (data not shown).

### Characteristics and detection rate of syphilitic rabbit blood.

In total, 50 syphilitic rabbits were included in this study, and the characteristics of the syphilitic rabbits are listed in Table 1. All 50 rabbits were serum T. pallidum particle agglutination (TPPA) test positive (100%), but 38 (76%) rabbits were toluidine red unheated serum test (TRUST) positive. Each syphilitic rabbit blood sample was divided into five parts (WB, WBL, plasma, serum, and BCL). Therefore, there were 250 samples in total. *polA* qPCR was used to detect T. pallidum DNA in these samples.

**TABLE 1:** Characteristics of the syphilitic rabbits included in this study^*a*^

Variable	Data
No. of rabbits	50
Duration of infection (days [mean ± SD])	9.60 ± 1.62
No. (%) of rabbits with more than 1 × 10^7^ treponemes/mL extracted from testes	34 (68.0)
No. (%) of positive serum TPPA tests	50 (100)
1/serum TPPA test titer (median [range])	1,280 (80–10,240)
No. (%) of positive serum TRUST	38 (76.0)
1/serum positive TRUST titer (median [range])	4 (1–64)

a TPPA, Treponema pallidum particle agglutination; TRUST, toluidine red unheated serum test.

As shown in Table 2, there was a significant difference in the positive detection rate of T. pallidum DNA between the WB group and other groups (*P < *0.001). The highest positive T. pallidum DNA detection rate was in the WBL group (82%). The positive T. pallidum DNA detection rates in the plasma, BCL, serum, and WB groups were 78%, 70%, 68%, and 6%, respectively. The positive T. pallidum DNA detection rate of the WB group was significantly lower than that of the other groups (*P < *0.001). In syphilitic rabbit blood samples that were TRUST negative, except those of the WB group that did not have detectable T. pallidum DNA, the detection rates of the WBL, plasma, serum, and BCL groups were 67%, 58%, 67%, and 50%, respectively. In syphilitic rabbit blood samples that were TRUST positive, the positive T. pallidum DNA detection rate of the WBL group was highest (86.8%), and the positive T. pallidum DNA detection rates of the plasma (84.2%), BCL (76.3%), serum (68.4%), and WB (7.9%) groups were lower.

**TABLE 2:** Detection rates for 50 syphilitic rabbits among five different sample types^*a*^

TRUST result	No. of rabbits	No. (%) of positive T. pallidum DNA results					*P*
		WB	WBL	Plasma	Serum	BCL	
Negative	12	0 (0)	8 (67.0)	7 (58.0)	8 (67.0)	6 (50.0)	
Positive	38	3 (7.9)	33 (86.8)	32 (84.2)	26 (68.4)	29 (76.3)	
Total	50^*b*^	3^*c*^ (6.0)	41 (82.0)	39 (78.0)	34 (68.0)	35 (70.0)	<0.001

a WB, whole blood; WBL, whole blood/lysed RBCs; BCL, blood cells/lysed RBCs.

b Blood samples from the 50 rabbits were all TPPA positive. The 50 peripheral blood samples from the rabbits were further divided into five groups, with each group being subdivided into 5 samples, for a total of 250 samples.

c Whole blood was significantly different from the other fractions (*P *< 0.001). There were no significant differences between any of the other groups (*P *> 0.05).

### Analysis of the copy number in syphilitic rabbit blood.

**FIG 3:**
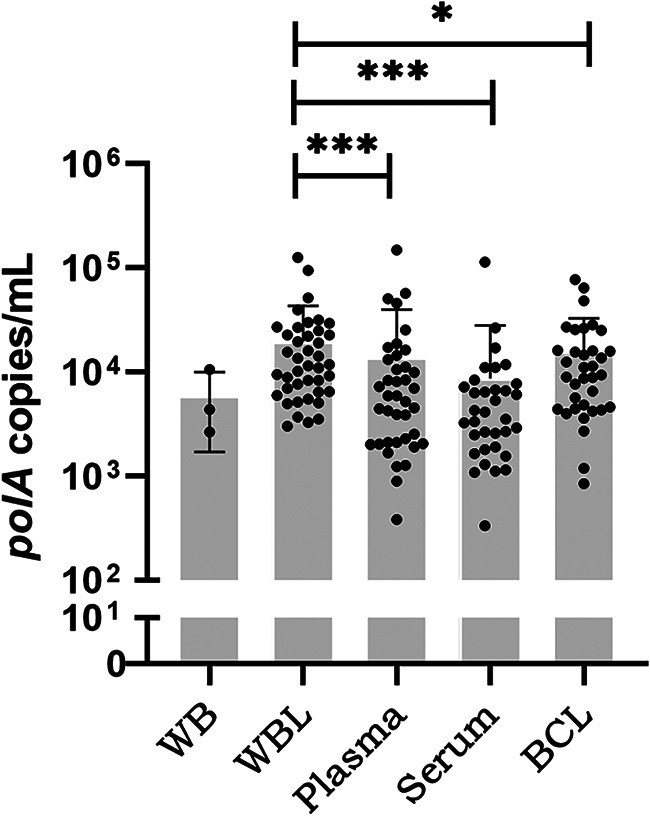
Copy numbers of all 152 *polA*-positive samples. Each dot represents a positive mean copy number of triplicates from one sample. Negative results were excluded. All data were converted to logarithms and analyzed. Significance was determined using the Wilcoxon matched-pairs test between WBL and other fractions. *, *P < *0.05; ***, *P < *0.001. Abbreviations: WB, whole blood; WBL, whole blood/lysed RBCs; BCL, blood cells/lysed RBCs.

The mean (±standard deviation [SD]) copy number determined from the *polA* assay was highest and most consistent in the WBL group (4.107 ± 0.383), and those in the BCL (3.987 ± 0.440), plasma (3.773 ± 0.543), and serum groups (3.617 ± 0.471) were lower (Fig. 3). Since the detection rate of the WB group was too low, its copy number was not statistically significant. When matched-pair samples were analyzed, most samples had similar changes in copy number among the five groups; that is, the copy number in the WBL group was significantly higher than those in the BCL, plasma, and serum groups (*P* < 0.05) (Fig. 3).

RBC lysis pretreatment significantly improved the detection rate of T. pallidum DNA in blood. Of the five sample types, WBL was the best for detecting blood T. pallidum DNA.

## DISCUSSION

In the present study, the qPCR assay we established had perfect amplification efficiency and could detect single DNA copies and 1 × 10^2^ treponemes/mL, as proven with plasmid DNA and T. pallidum DNA, respectively. We prepared serial dilutions of T. pallidum with five different media (NS, WB, WBL, plasma, and serum). The copy number of each medium was roughly the same at the initial concentration (1 × 10^5^ treponemes/mL), but the difference among the media began to appear as the concentration decreased. The NS and WB groups achieved detection limits of only 1 × 10^4^ treponemes/mL, for different reasons. According to a literature report (19), extracellular matrix components, such as fibronectin, collagens, and laminins, are also present in plasma and serum, and T. pallidum can adhere to these components. This adhesion may account for the observed disparity between the results obtained from NS, serum and plasma despite the absence of cells in three of them. In the WB group samples, which were viscous, a large number of cell fragments covered the spin column during DNA extraction. We speculated that the low concentration of T. pallidum could not be bound to the spin column, resulting in extraction failure (although a QIAamp DNA blood minikit was used, and the first incubation period was at least half an hour for full lysis). Moreover, there was also the possibility of hemoglobin inhibiting the PCR. Diluting the blood sample can mitigate clumping of cell fragments on the column, but it also reduces the detection sensitivity and may result in a detection limit of up to 10^2^ treponemes/mL (data not shown). However, at a T. pallidum concentration of only 10^2^ treponemes/mL in whole blood, a negative result can be obtained using the dilution method.

Aiming to solve the DNA extraction problem for whole blood, we pretreated the portion of simulated whole blood with RBC lysis buffer before DNA extraction, to remove RBCs, cell debris, and hemoglobin in advance. The results showed that the detection limit reached 1 × 10^2^ treponemes/mL, increasing by 2 orders of magnitude. This result indicates that the extraction efficiency of low-concentration T. pallidum DNA can be greatly improved by RBC lysis pretreatment, thus improving the positivity rate of whole-blood samples. During the RBC lysis process, high-speed centrifugation can reduce the loss of T. pallidum and concentrate a larger volume of blood, further improving the T. pallidum detection rate in whole blood with low concentrations of T. pallidum. Therefore, we recommend that when whole blood is used as a sample to detect T. pallidum DNA, RBCs be lysed before DNA extraction, to provide a stable and satisfactory result. In the plasma and serum samples, the detection limit of T. pallidum DNA reached 10^2^ treponemes/mL in the simulation medium, which was equivalent to that of the WBL samples.

Previous studies related to simulating blood samples have employed concentrations of T. pallidum higher than 10^6^ treponemes/mL (7, 14), which does not reflect the low concentrations of T. pallidum typically found in blood samples from syphilis patients. In fact, the copy number of T. pallidum in the peripheral blood of syphilis patients was generally lower than 10^3^ copies/mL (8, 12, 20), which is approximately 10^2^ treponemes/mL. In this study, we simulated a sample containing a lower range of treponemes, ranging from 10^3^ to 10^0^ treponemes/mL, which more closely reflects the clinical scenario. Therefore, after we verified the detection limit of different media in the simulation sample experiment, syphilitic blood was further explored. Blood samples were taken from syphilitic passaged rabbits (mostly ~8 to 12 days after infection) and divided into five parts, WB, WBL, plasma, serum, and BCL, for T. pallidum DNA detection. The results showed that among the five sample types, the detection rate and copy number of the WBL samples were highest and were significantly different from those of the other sample types. Notably, the detection rate in whole blood was very low but significantly increased after RBC lysis. When matched pairs were analyzed, the results of the WBL group tended to be more stable and higher than those of the plasma and serum groups. The WBL samples were mainly composed of leukocytes that T. pallidum could adhere to; thus, a relatively even distribution of T. pallidum cells was maintained in the WBL samples. While plasma and serum were acquired by low-speed centrifugation, which led to the sedimentation of T. pallidum, this redistribution made the T. pallidum loss in plasma and serum uncertain (14). These results demonstrate the advantages of the WBL method. The findings indicate that the concentration of T. pallidum cells was higher in blood cells than in plasma. This finding was also supported by the superior results obtained from the group in which samples were concentrated from BCL blood compared to those from the plasma samples. However, to our surprise, the results of the BCL group were worse than those of the WBL group. We theorized that the BCL group samples were more viscous than the WBL group samples, making RBC lysis insufficient, which resulted in a problem (column binding and hemoglobin inhibition) similar to that for whole blood. Therefore, under normal circumstances (volume of 200 μL for extraction), especially when the blood sample is less than 1 mL, pretreatment with RBC lysis buffer for whole-blood T. pallidum DNA detection is recommended to obtain satisfactory results. In addition, due to individual differences in the rabbits infected with T. pallidum, the onset time of orchitis was inconsistent. The earliest onset of orchitis in the 50 rabbits infected with T. pallidum was 8 days after infection with T. pallidum, and the latest onset of orchitis was 12 days after infection. All rabbits with T. pallidum infection had TPPA-positive results. Because the Treponema antibody was produced earlier than the non-Treponema antibody, TPPA-positive and TRUST-negative results occurred. Therefore, 12 rabbits had a negative TRUST test. However, of these TRUST-negative rabbit blood samples, some tested positive for T. pallidum DNA using our method. This suggests that the PCR method can play a role in the diagnosis of syphilis.

There were some limitations in our study. Further study is needed to understand the reason for the low detection limit in the NS group. It is worth exploring the detection limit of T. pallidum in the PBMCs of syphilis patients further. The current study reflects T. pallidum DNA only in an *in vitro* experimental system and in syphilitic rabbit blood. A study with a large sample size of clinical samples should be conducted in the future. Furthermore, although removing RBCs could reduce the hemoglobin inhibition of T. pallidum PCR tests in blood, the large amount of host genome DNA might still inhibit the reaction. Finally, our PCR detection system used the standard T. pallidum strain Nichols as a positive quality control, and at the same time, we used the human RNP gene as a housekeeping gene to monitor the sampling and PCR process. However, when testing samples from rabbits, we only used the standard T. pallidum strain Nichols as the quality control and did not introduce rabbit-derived RNP genes as housekeeping genes.

In conclusion, T. pallidum DNA detection is more successful in whole blood/lysed RBCs than in whole blood, plasma, serum, or BCL. We suggest that blood T. pallidum DNA extraction be performed with RBC lysis pretreatment, which could significantly improve the yield of T. pallidum DNA.

## MATERIALS AND METHODS

### Preparation of T. pallidum.

The Nichols strain of T. pallidum were serially passaged by intratesticular inoculation of New Zealand white rabbits, as previously described (21). T. pallidum cells were extracted by mincing and shaking the testes in normal saline (NS). Then, the T. pallidum count was enumerated by dark-field microscopy and adjusted with NS to 1.0 × 10^6^ treponemes/mL. The animal experiments followed protocols approved by the experimental animal ethics committee of the School of Medicine, Xiamen University.

### *Ex vivo* blood spiking experiment.

To explore the detection limit of T. pallidum in different blood fractions *ex vivo*, 20 mL of blood was collected from a healthy volunteer, utilizing two nonanticoagulant collection tubes with a volume of 4 mL each and six anticoagulant collection tubes containing EDTA and having a volume of 2 mL each. Subsequently, the blood was fractionated into three components, whole blood, plasma, and serum. A sample (1.0 × 10^6^ treponemes/mL) was diluted with NS to 1.0 × 10^5^ treponemes/mL, which was the same concentration as whole blood, plasma, and serum, and serially 10-fold diluted with the same medium to 1.0 × 10^0^ treponemes/mL. Then, 0.2 mL of the whole blood was subjected to RBC lysis pretreatment. In brief, five kinds of simulation media (NS, whole blood, whole blood/lysed RBCs, plasma, and serum) were prepared with ~1.0 × 10^5^ to 1.0 × 10^0^ treponemes/mL, and a set of five simulation media, bereft of T. pallidum supplementation, was employed as a negative control in the study. Each medium had seven samples. DNA was extracted after all samples were prepared.

Two additional types of whole-blood dilution experiments were conducted in this study. The first experiment involved serial dilution of simulated whole blood with NS (SBN), where 10^6^ treponemes/mL of simulated whole blood were subjected to 10-fold serial dilution with NS, resulting in a final concentration of 10^0^ treponemes/mL. This experiment ensured the simultaneous dilution of T. pallidum and blood cells, maintaining their relative proportions unchanged. The second experiment involved whole blood diluted with NS (BDN), where each sample of the whole-blood group was separately diluted 10-fold with NS. In this case, T. pallidum cells were subjected to serial dilution while maintaining the blood cells at a constant 10% of the whole-blood sample. DNA extraction in both experiments was carried out without additional steps.

### Collection and processing of syphilitic blood.

Fifty syphilitic rabbits were included in this study. When the syphilitic rabbits exhibited orchitis (usually ~8 to 12 days after infection), 4 mL of blood was drawn from the heart and was equally distributed between a nonanticoagulant collection tube and an EDTA tube. Two 200-μL portions of EDTA-treated blood were transferred into Eppendorf tubes: one was used as the whole-blood (WB) sample, and the other was pretreated with RBC lysis and used as the whole-blood/lysed RBC (WBL) sample. Plasma was separated from the remaining EDTA-treated blood by centrifugation at 3,000 × *g* for 5 min. All plasma was placed into an Eppendorf tube and used as the plasma sample. The remaining blood without plasma was treated with lysis buffer and used as the blood cell/lysed RBCs (BCL) group. Serum was obtained from the nonanticoagulant collection tube by centrifugation at 3,000 × *g* for 5 min at room temperature, and these samples were considered the serum group. Part of the serum was used to determine titers with the T. pallidum particle agglutination (TPPA) test and toluidine red unheated serum test (TRUST). The blood components were collected and subsequently stored at 4°C. Within 6 h of collection, separation was carried out, followed by storage at −20°C for DNA extraction purposes.

### Red blood cell lysis pretreatment.

Anticoagulated blood (WBL and BCL) samples requiring pretreatment with RBC lysis were subjected to treatment with RBC lysis buffer (Solarbio, Beijing, China; R1010; 500 mL) containing ammonium chloride, in accordance with the manufacturer’s provided instructions. First, 200 μL of blood was added to 600 μL of RBC lysis buffer in a tube and gently inverted several times. Then, the tubes were placed on ice for 15 min. During that period, the tubes were inverted two times for enhanced lysis. Next, the tubes were centrifuged at 20,000 × *g* for 10 min at 4°C; the supernatant was carefully discarded, and the pellet was resuspended in another 400 μL of RBC lysis buffer. After sufficient vortexing, the tubes were again centrifuged at 20,000 × *g* for 10 min at 4°C. Finally, the supernatant was fully discarded, and the pellet was resuspended in 200 μL of NS for DNA extraction.

### DNA extraction.

DNA was extracted from 200 μL of each sample type (NS, WB, WBL, plasma, serum, and BCL). A QIAamp DNA blood minikit (Qiagen, Valencia, CA; 51306) was used according to the manufacturer’s instructions, with the following modifications. Initially, the samples were subjected to a 30-min incubation period in buffer AL and proteinase K, with periodic inversion of the tubes to ensure proper mixing; DNA was eluted from the Qiagen columns in 100 μL of elution buffer at 65°C. Extracted DNA was stored at −20°C. New aliquots of reagent were used, and a negative extraction control (NS) was included in each batch.

### Development of TaqMan-based real-time qPCR testing for the detection of the *polA* gene.

The primers and probe sequences of the *polA* gene (GenBank accession number U57757.1) used in this study are listed in Table 3. The primers and probe were synthesized by Sangon Biotech (Shanghai, China). The qPCR assay was performed in a 25-μL reaction volume containing 5 μL of template DNA, 1 U of Hot-Start *Taq* polymerase (TaKaRa, Dalian, China; R007B), 4.5 mM MgCl_2_ (Promega, Beijing, China; A3513), and optimized concentrations of the primers and probe (Table 3). Diethyl pyrocarbonate (DEPC)-treated water was used as a negative reagent control, and NS was used as a negative extraction control. Additionally, 10^3^ and 10^0^ copies/μL pET-24a^+^ plasmids were used as the positive control and weakly positive control, respectively. All samples were tested in triplicate. Real-time qPCR was performed on the Roche LightCycler 480 II system with the following conditions: 95°C for 3 min, then 45 cycles of 95°C for 10 s and 60°C for 30 s (signal acquisition), and finally, 37°C for 10 s.

**TABLE 3:** Sequences and concentrations of the *polA* primers and probe used in this study

Primer/probe^*a*^	Sequence (5′–3′)	Concn (μM)	Amplicon length (bp)
*polA*-F	TACGGTGCAAGTGCTCAGAC	0.2	143
*polA*-R	CAGGCACATTGTCGGAGGAA	0.2	
*polA*-P	HEX-TTGCGACGCTGCGTACGTACAGCAACGGT-BHQ-1	0.12	

a F, forward primer; R, reverse primer; P, probe.

The *polA* qPCR assay results were considered positive when the fluorescent signal was clearly visible and the crossing point (Cp) value was lower than 38. In order to prevent false-negative results, samples with Cp values between 38 and 40 were subjected to a retest, with a positive result being recorded if the Cp value was ≤38 on the second test and a negative result being recorded otherwise. Other than the circumstances above, the qPCR assay result was considered negative. The Cp values of positive samples were converted into copy numbers according to a standard curve.

### Preparation of the standard curve, limit of assay detection, and assay specificity.

The PCR product of *polA* was cloned into the pET-24a^+^ cloning vector (synthesized by Tsingke Biotechnology, Beijing, China). The gene copy numbers were calculated from the concentration of the plasmid DNA, as determined using a Qubit double-stranded DNA (dsDNA) high-sensitivity (HS) assay kit (Thermo Fisher Scientific, Shanghai, China; Q33230). Tenfold serial dilutions of the plasmid DNA were used to generate a standard curve ranging from ~10^6^ to 10^0^ copies/μL DNA for the *polA* gene.

In addition, total DNA was extracted from fresh samples at 1.0 × 10^7^ treponemes/mL using a QIAamp DNA blood minikit, as described above. The initial sample volume of 200 μL and elution volume of 200 μL elution buffer produced a concentration of 1 × 10^7^ treponemes/mL. Serial 10-fold dilutions of ~10^1^ to 10^7^ were made using Tris-EDTA water, which resulted in serial dilution samples of ~1 × 10^7^ to 1 × 10^0^ treponemes/mL. The limit of detection of the assay was determined using serial dilutions of T. pallidum DNA.

During assay development, the identity of the *polA* PCR product was confirmed using both agarose gel electrophoresis (3% agarose) and DNA sequencing (Sangon Biotech), including for some weakly positive samples. Nucleic acids of other sexually transmitted organisms, including Chlamydia trachomatis, *Herpes simplex virus* (HSV), *Human papillomavirus* (HPV), and Neisseria gonorrhoeae, and other human DNA and rabbit DNA were obtained from clinical samples and used to test the cross-reactivity of the *polA* qPCR assay.

### Statistical analysis.

Fisher’s exact test (or the chi-square test) were used to compare differences in detection rate. The Wilcoxon matched-pairs test and the Mann-Whitney U test were used to determine differences between the median log_10_ copy number in the blood groups (WB, WBL, plasma, serum, and BCL) following the *in vitro* spiking experiments and between the paired blood fractions. The analyses were carried out using GraphPad Prism 8.0 (GraphPad Software, San Diego, CA) and SPSS 25.0 (IBM SPSS, Chicago, IL, USA). *P < *0.05 was considered statistically significant.

### Data availability.

The data supporting the conclusions of this article will be made available by the authors, without undue reservation.
